# Supplementary material for: Selectivity of Hydroxamate- and Difluoromethyloxadiazole-Based Inhibitors of Histone Deacetylase 6 In Vitro and in Cells
Source: Int J Mol Sci. 2023 Mar 1;24(5):4720. doi: 10.3390/ijms24054720 (PMC10003107; doi:10.3390/ijms24054720)

# Selectivity of hydroxamate- and difluoromethyloxadiazole-based inhibitors of histone deacetylase 6 *in vitro* and in cells

**Jakub Ptacek[1], Ivan Snajdr[2], Jiri Schimer[2], Zsofia Kutil[1], Jana Mikesova[1], Petra Baranova[1], Barbora Havlinova[1], Werner Tueckmantel[3], Pavel Majer[2], Alan Kozikowski[3],[4] and Cyril Barinka[1] ***

[1]*Institute of Biotechnology CAS, BIOCEV, Prumyslova 595, 252 50 Vestec, Czech Republic*

[2]*Institute of Organic Chemistry and Biochemistry of the Academy of Sciences of the Czech Republic, Flemingovo n. 2, 166 10 Prague 6, Czech Republic*

[3] *StarWise Therapeutics LLC, University Research Park, Inc., Madison, WI 53719, United States*

[4] *Department of Medicinal Chemistry and Pharmacognosy, College of Pharmacy, University of Illinois at Chicago, Chicago, IL 60612, United States*

## Supplementary information:

**Supplementary Table 1:** List of HDAC genes, their source, and primers used. HDAC genes were obtained from listed sources, and the sequences encoding amino acid ranges given in the "AAs" column were amplified using primers listed. All genes were cloned to expression plasmids using the Gateway cloning system.

| Gene | AAs | Source | Primer sequence, 5'–3' |
|---|---|---|---|
| HDAC1 | 1-482 | Addgene | Fw1: GAGAACCTGTACTTCCAGGGCGGAGGCaccATGGCGCAGACGCAGGGCACCCGGAGG<br>Rev: GGGGACCACTTTGTACAAGAAAGCTGGGTTATTAGGCCAACTTGACCTCCTCCTTGACC |
| HDAC2 | 1-488 | GenScript | Fw1: GAGAACCTGTACTTCCAGGGCGGAGGCACCATGGCGTACAGTCAAGGAGGCGGC<br>Rev: GGGGACCACTTTGTACAAGAAAGCTGGGTTATTAGGGGTTGCTGAGCTGTTCTGATTTGG |
| HDAC3 | 1-428 | Addgene | Fw1: GAGAACCTGTACTTCCAGGGCGGAGGCACCATGGCCAAGACCGTGGCCTATTTCTAC<br>Rev: GGGGACCACTTTGTACAAGAAAGCTGGGTTATTAAATCTCCACATCGCTTTCCTTGTC |
| HDAC4 | 1-1084 | Addgene | Fw1: gagAACCTGTACTTCCAGGGCGGAGGCACCATGAGCTCCCAAAGCCATCCAGATG<br>Rev: GGGGACCACTTTGTACAAGAAAGCTGGGTTATTACAGGGGCGGCTCCTCTTCCATGGG |
| HDAC5 | 679-1095 | Addgene | Fw1: GAGAACCTGTACTTCCAGGGCGGAGGCACCATGAACTCTCCCAACGAGTCGGATGGG<br>Rev: GGGGACCACTTTGTACAAGAAAGCTGGGTTATTACAGGGCAGGCTCCTGCTCCATGGG |
| HDAC6 | 2-1215 | Open Biosystems | Fw1: GAGAACCTGTACTTCCAGTCTATGACCTCAACCGGCCAGGATTCCAC<br>Rev: GAGGATATGCCCCACCCACACTAATAACCCAGCTTTCTTGTACAAAGTGGTCCCC |
| HDAC7 | 1-915 | Addgene | Fw1: gagAACCTGTACTTCCAGGGCGGAGGCACCATGgacCTGCGGGTGGGCCAGcgg<br>Rev: GGGGACCACTTTGTACAAGAAAGCTGGGTTATTAGAGATTCATAGGTTCTTCCTCCTC |
| HDAC8 | 1-377 | M. Schutkowski, Martin Luther University Halle | Fw1: TCGGAGAACCTGTACTTCCAGTCTACCATGgaggagccggaggaaccggcgg<br>Rev: GGGGACCACTTTGTACAAGAAAGCTGGGTTATTAagaccacatgcttcagattccctttg |
| HDAC9 | 1-1069 | D. Mauceri, University of Heidelberg | Fw1: gagAACCTGTACTTCCAGGGCGGAGGCACCATGcacAGTATGATCAGCTCAgtg<br>Rev: GGGGACCACTTTGTACAAGAAAGCTGGGTTATTACAAGGCTGGCTCCTCTTCCATAGG |
| HDAC10 | 1 – 667 | Open Biosystems | Fw1: tcggagaacctgtacttccagtctgggaccgcgcttgtgtaccatgagg<br>Rev: ggggaccactttgtacaagaaagctgggttatcaagccaccaggtgaggatggcac |
| HDAC11 | 1-346 | E. Seto, The George Washington University | Fw1: gagAACCTGTACTTCCAGGGCGGAGGCACCATGctacacacaacccagctgtac<br>Rev: AGACTGGAAGTACAGGTTCTCAGAACCGGGCACTGCAGGGGGAAGCAG |

**Supplementary Table 2:** Inhibition potency of compounds tested on a panel of HDACs using peptidic substrates. $IC_{50}$s are listed in nM concentration ± standard deviation.

| Compound | $IC_{50}$ [nM] | | | | | | | | | | |
|---|---|---|---|---|---|---|---|---|---|---|---|
| | HDAC1 | HDAC2 | HDAC3 | HDAC4 | HDAC5 | HDAC6 | HDAC7 | HDAC8 | HDAC9 | HDAC10 | HDAC11 |
| **Next A** | 362 ± 119 | 276 ± 95.5 | 1419 ± 145 | 14773 ± 1687 | 6616 ± 2663 | 3.8 ± 2.1 | 2432 ± 298 | 1544 ± 757 | 2000 ± 769 | 11.0 ± 9.1 | 10601 ± 2214 |
| **ACY-775** | 1194 ± 93 | 1594 ± 449 | 10800 ± 1549 | 7289 ± 1561 | 1741 ± 421 | 1.11 ± 0.55 | 7864 ± 2444 | 8993 ± 411 | 20585 ± 686 | 34.0 ± 7.8 | >50000 |
| **ACY-1215** | 106 ± 43 | 69.7 ± 16.9 | 153 ± 20 | 19355 ± 3118 | 3244 ± 537 | 2.37 ± 0.37 | 8771 ± 3732 | 1072 ± 552 | >50000 | 18.2 ± 1.2 | 3806 ± 2710 |
| **TSA** | 0.71 ± 0.20 | 1.81 ± 0.83 | 6.92 ± 0.80 | 8477 ± 280 | 2331 ± 335 | 0.41 ± 0.04 | 3255 ± 499 | 909 ± 127 | 18240 ± 283 | 4.3 ± 3.5 | 4492 ± 1418 |
| **Tub A** | 2288 ± 172 | 2545 ± 665 | 8237 ± 1617 | 5120 ± 698 | 2700 ± 220 | 4.65 ± 0.49 | 570 ± 169 | 4527 ± 2211 | 4510 ± 772 | 3.8 ± 3.1 | 38372 ± 6458 |
| **Tubacin** | 102 ± 13 | 296 ± 160 | 4172 ± 602 | > 50,000 | 33535 ± 3726 | 35.9 ±9.7 | >50,000 | 5367 ± 334 | >50000 | 678 ± 24 | 6548 ± 3720 |
| **6** | 49.2 ± 15.2 | 47.6 ± 0.1 | 91.2 ± 15.3 | 4442 ± 449 | 1930 ± 313 | 0.35 ± 0.01 | 2823 ± 102 | 1726 ± 405 | 5734 ± 551 | 68.3 ± 18 | >50000 |
| **7** | >50000 | >50000 | >50000 | >50000 | >50000 | 2.06 ± 0.39 | >50000 | >50000 | >50000 | >50000 | >50000 |

**Supplementary Figure S1:** Expression plasmid for full-length human HDAC6. An example of plasmid used for expression of HDACs in mammalian HEK293T cells. HDACs sequences are N-terminally fused to the TEV-cleavable TwinStrep-FLAG-HALO tag.

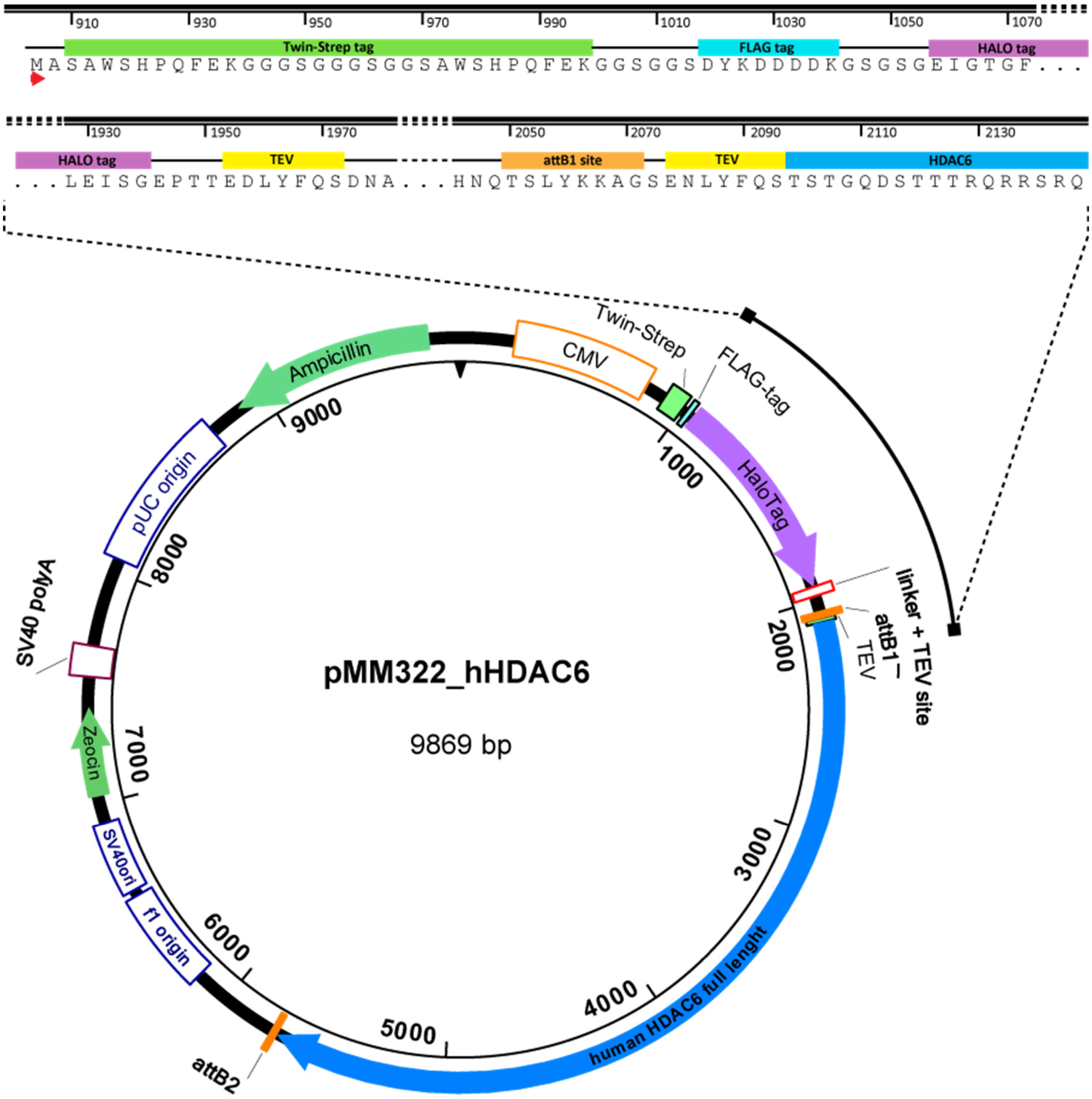

**Supplementary Figure S2:** Western blots used for quantification of tubulin acetylation. Three different antibodies were used to label tubulin (TUB; total protein control, used for normalization), acetylated tubulin Lys-40 (Ac-TUB), and acetylated histone H3 (Ac-HisH3; acetylation status of histone – non-specific inhibition of HDACs deacetylating histones)

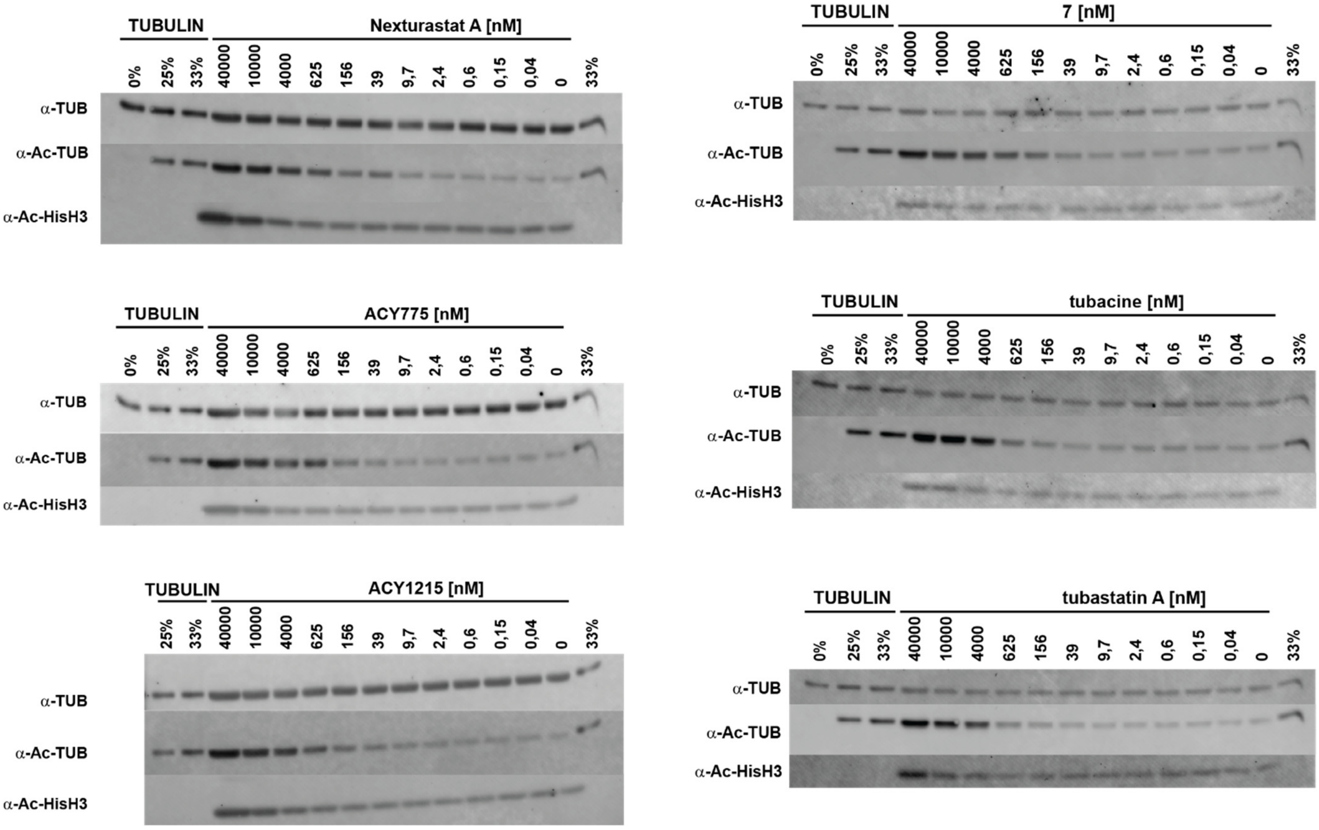

Supplement: Supplementary file 1 [file ijms-24-04720-s001.zip › ijms-2234257-supplementary.pdf]
